# The Influence of High Physical Demand on the Occurrence of Major Muscle and Ligament Injuries in Professional Soccer Athletes: A Systematic Review

**DOI:** 10.1055/s-0044-1786171

**Published:** 2024-05-13

**Authors:** Matheus Martins Godoy, Lucas Ferreira Gonçalves, Thiago da Mata Martins, Renato Ventura

**Affiliations:** 1 Curso de Medicina, Centro Universitário de Patos de Minas, (UNIPAM), Patos de Minas, MG, Brasil

**Keywords:** athletes, knee injuries, muscle, skeletal, soccer/injuries

## Abstract

**Objective** The present systematic review aimed to investigate the influence of high physical demand on the increase in muscle and ligament injuries in professional soccer athletes.

**Methods** We analyzed scientific publications to determine the incidences of the main injuries, their causes and mechanisms, and their association with high physical demand. We compared amateur and professional players and assessed the effectiveness of FIFA11+ as a prevention alternative. Searches occurred on Scielo, Pubmed, and Google Scholar databases. The filters were the topic, publication date (last 5 years), and study relevance. The indexing terms were the following: *Overuse* , *Calendar* , *Injuries* , *Muscular* , *Ligament* , *Athletes* , *Soccer* , *Football* . We described the main data obtained to compare and analyze the results. This study complied with the Preferred Reporting Items for Systematic Reviews and Meta-Analyses (PRISMA) statement guidelines.

**Results** The query resulted in 24 articles published from 2019 to 2023. The high physical demand increased the risk of injuries. Most injuries occurred in the lower limbs. The most common injuries were strains, sprains, contractures, and ligament ruptures. FIFA11+ presented itself as a viable prevention alternative.

**Conclusion** We concluded that high physical demand increases the most frequent muscle and ligament injuries in professional soccer players, that is, strains, sprains, contractures, and ligament rupture, suggesting the FIFA11+ program as a prevention alternative.

## Introduction

Soccer is one of the most popular sports worldwide. It is played in several countries by individuals from all age groups and both genders. However, professional soccer players present a high incidence of injuries due to the intense physical demand in training and matches throughout the year. 1 2

The incidence of sports injuries and their risk factors is a subject of intense debate. The search for good performance and success in sports causes many players to undergo threshold physical effort or beyond their physiological limits, resulting in overload-related injuries. 3 Soccer requires a lot of impact from physical contact, short, fast, and discontinuous movements such as acceleration and deceleration, jumps, and sudden directional changes. 1 4

It is estimated that soccer accounts for 50 to 60% of sports injuries affecting different muscle groups, mainly in the lower limbs. 2 Injuries often negatively impact not only the player's quality of life but the team's performance, resulting in strategic and economic losses to the club due to the absence in training and matches. 5

Taking care of the athlete's health and implementing injury prevention strategies are critical to maximize the team's chance of success and the player's professional growth. Therefore, knowledge of injuries emerges as a strategy for developing plans to prevent or minimize them in sports, reducing the losses and unhappiness resulting from players' absence. 6 7

The growth of national and international competitions, especially with the increase in the number of official matches, makes it critical to know about the different types of injuries that affect soccer players the most with the goal of prophylaxis as well as taking care of the athlete's health and safety. ^6^ Therefore, this study aimed to investigate and address the influence of high physical demand on the incidence of the main muscle and ligament injuries in professional soccer players.

## Materials and Methods

We conducted this study according to the guidelines for systematic reviews, whose goal is to examine and discuss articles published on a determined topic. 8 The Preferred Reporting Items for Systematic Reviews and Meta-Analyses (PRISMA) guidelines were followed.

The present study is a systematic review. The review had six stages: 1) identification of the topic and selection of the research question; 2) establishment of criteria for literature search and study inclusion and exclusion; 3) definition of the information to be extracted from the selected studies; 4) study categorization; 5) evaluation of studies included in the integrative review and their interpretation; and 6) review presentation.

The first stage used the Patient, Intervention, Comparison, and Outcome (PICO) strategy to define the following research question: “Does high physical demand have an influence in the increase of major muscle and ligament injuries occurrence in professional soccer players?” In this question, P refers to professional soccer players, I is high physical demand, C is regular physical demand, and O refers to increased occurrence.

We searched for articles about the intended outcome to answer this question using the terminologies registered in the Health Sciences Descriptors (DeCS) created by the Virtual Health Library and developed by the Medical Subject Headings of the U.S. National Library of Medicine, allowing the use of common terminology in Portuguese, English, and Spanish. The descriptors included *Overuse* , *Calendar* , *Injuries* , *Ligament* , *Muscular* , *Athletes* , *Football* , and *Soccer* . Keyword crossing used the Boolean operators *and* , *or* , and *not* .

The bibliographic survey occurred through electronic searches in the following databases: Scientific Electronic Library Online (SciELO), National Library of Medicine (PubMed), and Google Scholar.

The search took place in September 2023. For inclusion criteria, there was no language limitation, considering articles in English and Portuguese published in the last 5 years (from 2019–2023) about the topic and available electronically in its full format. Articles that did not meet the inclusion criteria were excluded.

## Results

**Fig. 1:**
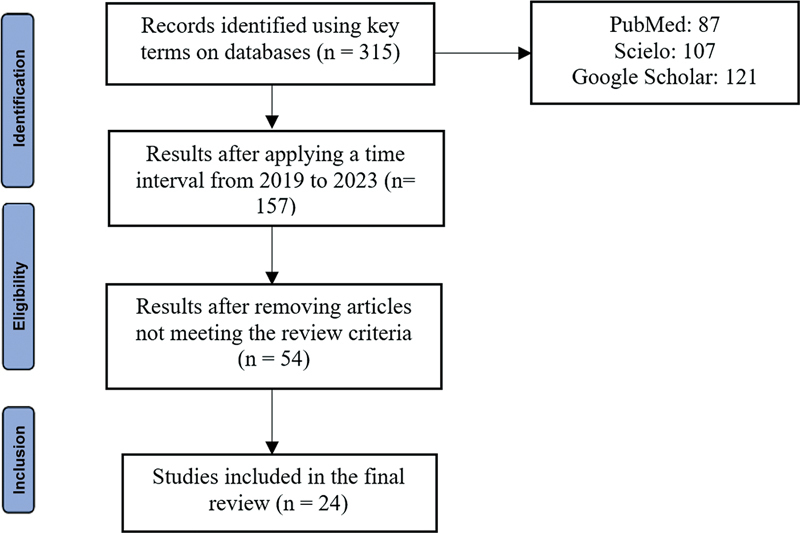
Study selection flowchart.

We identified a total of 315 studies. The time interval filter (2019–2023) application resulted in 157 articles for further investigation, according to the established exclusion criteria, generating a sample of 54 publications for complete reading. Of these 54 studies, we excluded 30 because they were irrelevant to the present study. The final sample for this review consisted of 24 articles. The flowchart ( Fig. 1 ) demonstrates this paper selection process.

We tabulated the selected studies ( Table 1 ) 5 6 8 9 10 11 12 13 14 15 16 17 18 19 20 21 22 23 24 25 26 27 28 29 including authors' names and year of publication, type of article, title, study objective, and main findings.

**Table 1:** Articles included in the systematic review

#	Author and year	Article type	Title	Main findings
1	Drummond et al., 6 2021	Prospective study	Incidence of injuries in soccer players – mapping foot: a prospective cohort study	In a soccer tournament, 86.9% of injuries in male players occurred in the lower limbs. Stretch injuries were the most common and mainly affected the semitendinosus, biceps femoris, and gastrocnemius muscles.
2	Marques, 5 2021	Systematic review	Injuries in Portuguese female soccer athletes	In female soccer athletes, 85.5% of injuries affected the lower limbs.
3	Lima et al., 8 2022	Systematic review	Injuries in soccer athletes: a theoretical study	Lower limb injuries are more frequent in soccer due to the demands imposed on this anatomical region.
4	Castelo et al., 9 2022	Systematic review	Injuries in professional soccer: a systematic review	Stretch injuries are the most common and mainly affect the semitendinosus, biceps femoris, and gastrocnemius muscles. Muscle contracture damages the semitendinosus, semimembranosus, and biceps femoris muscles. Fatigue is the main cause of muscle contracture, which greatly affects elite soccer players.
5	Silva et al., 10 2019	Systematic review	Incidence of musculoskeletal injuries in professional soccer players in Brazil	Stretch injuries are the most common and affect the semitendinosus, biceps femoris, and gastrocnemius muscles. Stretching refers to an excessive fiber elongation and occurs during sudden acceleration, deceleration, or sprints.
6	Teixeira et al., 11 2021	Field research	Epidemiological analysis of injuries in professional football athletes in two football clubs in Goiânia, Goiás, Brazil	Stretch injuries are the most common and affect the semitendinosus, biceps femoris, and gastrocnemius muscles.
7	Allah, 12 2022	Systematic review	Physical therapy for ankle sprain injuries in soccer players: a literature review	A sprain is an injury to joint ligaments, and it commonly affects the ankle, with potential partial or complete rupture. It results from sudden movements, such as plantar flexion with foot inversion.
8	Ribeiro et al., 13 2022	Systematic review	The physiotherapeutic approach to lateral ankle sprain: a literature review.	A sprain is an injury to joint ligaments, and it commonly affects the ankle, with potential partial or complete rupture. It results from sudden movements, such as plantar flexion with foot inversion.
9	Meneses, 14 2021	Systematic review	Sports injuries in futsal athletes: a systematic review of the literature	In soccer, knee sprains frequently result from a sudden directional change in the knee with the foot in a fixed position. Capsuloligamentous and meniscal injuries stand out.
10	Feitoza Neto et al., 15 2021	Systematic review	Injuries to knee structures in soccer players	In soccer, knee sprains frequently result from a sudden directional change in the knee with the foot in a fixed position. Capsuloligamentous and meniscal injuries stand out.
11	Farias, 16 2021	Systematic review	Applicability of strength exercises in the prevention and rehabilitation of grade I and II muscle injuries in elite soccer players	Contracture results from incorrect muscle contraction with no return to the regular relaxed state. The overload of anaerobic respiration leads to lactic acid accumulation, causing this phenomenon. Muscle contracture mainly damages the semitendinosus, semimembranosus, and biceps femoris muscles. Fatigue is its main cause, explaining why these injuries are common in elite soccer players.
12	Zhang and Wanh, 17 2023	Field research	Sports injuries in professional soccer players	Muscle contracture damages the semitendinosus, semimembranosus, and biceps femoris muscles. Fatigue is its main cause, explaining why these injuries are common in elite soccer players.
13	Silva et al., 18 2020	Systematic review	Physical therapist's role with players with anterior cruciate ligament injuries	Ligament tears depend on the performed movement. Athletes frequently present anterior cruciate ligament injuries due to excessive force during knee rotation.
14	Borba, 19 2021	Systematic review	Criteria for return to soccer after anterior ligament reconstruction surgery: a literature review	Ligament tears depend on the performed movement. Athletes frequently present anterior cruciate ligament injuries due to excessive force during knee rotation.
15	Sousa, 20 2021	Systematic review	Plyometrics in the prevention of anterior cruciate ligament injuries in soccer athletes: an integrative literature review	Knee ligament injuries can cause loss of integrity and functionality in the short and long term and be irreversible, resulting in the retirement of soccer players.
16	Choriyev, 21 2021	Field research	Planning and organizing training of football players	We must consider the relatively low functional capacity of children aged 8 to 11. These children required training in age-specific ball techniques and game tactics. For teenagers aged 12 to 17, the focus changes to individual preparation, with more elaborate exercises emphasizing movement speed, resistance, agility, and flexibility in different age groups. Therefore, we must select proper exercises based on the match, their influence on the body's functions, and their duration and intensity.
17	Jones et al., 22 2019	Systematic review	Injury incidence, prevalence, and severity in high-level male youth football: a systematic review	Age influences the total incidence of injuries in athletes. Older players have higher injury incidence rates. One-fifth of injuries are severe and can cause the athlete to stop playing for at least 28 days, which harms the subject and the team, as both lose out in seasonal development.
18	Pfirrmann et al., 23 2016	Systematic review	Analysis of injury incidences in male professional adult and elite youth soccer players: a systematic review	Injury rates were higher in matches than in training both for young and adult players. Youth athletes had a higher incidence of training injuries than professional players.
19	Cezarino et al., 24 2020	Prospective study	Injury profile in a Brazilian first-division youth soccer team: a prospective study	The injury incidence was higher in matches than in training, and the oldest age group (sub-20) had the highest injury incidence rate in games, while the sub-17 group had the highest injury incidence rate in matches. Injury incidence rates for players under 20 years old are close to professional players.
20	Yang et al., 25 2022	Systematic review and meta-analysis	Effects of the “FIFA11+ Kids” program on injury prevention in children: a systematic review and meta-analysis	FIFA11+ significantly decreased the risk of overall injuries compared to regular warm-up training. Soccer players aged under 15 are at the highest risk of injuries among all age groups analyzed as they have a lower level of physical development. FIFA11+ Kids is effective in preventing soccer-related injuries in young players. The program can significantly reduce the risk of ankle, knee, and lower limb injuries.
21	Al Attar et al., 26 2022	Clinical trial	The FIFA 11+ kids injury prevention program reduces injury rates among male children soccer players: a clustered randomized controlled trial	FIFA11+ significantly reduced knee, leg, and ankle injuries, the likelihood of a player becoming injured, and the rates of contact, non-contact, and overuse injuries. Therefore, this study confirms the critical role of implementing the program in younger players to prevent injuries.
22	Pinto et al., 27 2021	Systematic review	FIFA 11+ for injury prevention in soccer players: a systematic review	In addition to all the indications from Al Attar's study, the prevention effect is higher when all team players adhere to the program. When targeted at high-competition athletes, outcomes are less significant due to periodization and competition calendar. Furthermore, the arrival of players from other clubs in midseason reduces the program's effectiveness, as do players who fail to complete the protocols due to injury or illness. The program also improves performance variables, causing significant improvements in dynamic postural control.
23	Materne et al., 28 2020	Prospective study	Injury incidence and burden in a youth elite football academy: a four-season prospective study of 551 players aged from under 9 to under 19 years	Compared to other age groups, sub-16 and sub-18 players had higher incidence rates of overuse injuries. In the remaining players, bruises, sprains, and growth-related injuries were the most common. Meniscus/cartilage injuries were the most severe lesions.
24	Aiello et al., 29 2022	Systematic review	Injury-inciting activities in male and female football players: a systematic review	High-intensity running and kicking activities may be the main triggers for thigh and groin injuries, while contacts may be the most common triggers for ankle injuries. Contact and pressure may be the most common inciting activities leading to anterior cruciate ligament injuries, but there is no consensus in the literature.

Of the 24 articles selected, 16 were systematic reviews, 3 were field research, 3 were prospective studies, 1 was a systematic review and meta-analysis, and 1 was a clinical trial. However, they all address the main theme: the occurrence of sports injuries in soccer players. The authors' main objectives included investigating the prevalence of different types of injuries (looking for the most common ones), the different incidences of injuries in amateur (base categories) and professional players, the effectiveness of the FIFA11+ program as a preventive alternative and understanding the influence of risk factors and mechanisms for the occurrence of certain types of injury.

## Discussion

### Influence of High Physical Demand

#### Functional Capacity

The functional capacity of the player is paramount because of the significant difference between professional and amateur athletes. Each age group requires different training strategies, with distinct loads, intensity of physical activity, exercise duration, and appropriate selection for each category.

The stratification of the sport modality and the variables from each athlete provide indications, such as ball techniques and game tactics, for players aged 8 to 11 years old, and individual preparation with an emphasis on movement speed, resistance, agility, and flexibility for players aged 12 to 17. This stratification highlights the role of grading intensity as age advances to preserve the athlete's physical health up to the professional level. Otherwise, the lack of planning, with exaggerated demands, may expose these players to greater chances of injuries. 21

#### Comparison of Amateur and Professional Players

Analyzing the incidence rate of injuries in high-level youth soccer, we noted an influence of advancing age, as older athletes have higher incidence rates. Among these injuries, 20% are severe, and result in absence for at least 28 days. 22

This analysis is consistent with the fact that professional match calendars are more competitive and require greater physical demand compared with base (amateur) athletes. The higher frequency of short breaks between official matches, training intensity, time zone variation affecting rest, and the distinct climates from different locations, among other details, generate a set of factors contributing to higher physical wear and tear in professional players.

Younger athletes from base categories have a less intense calendar, with greater chances of preventing overload-related muscle and ligament injuries, partially explaining the different injury incidences. However, when comparing younger players from sub-9 and sub-19 categories, the latter presented high injury incidence rates due to the increasing training intensity and frequency of championship matches. Therefore, we infer that the higher intensity and match frequency, with no proper physical preparation, are risk factors for injuries even in young athletes. 28

Furthermore, this data demonstrates that the transition from amateur (base categories) to professional category, following the same notion of advancing age, could be a risk factor for the higher incidence rate in these athletes.

#### Training and Matches

The high physical demand for high-performance athletes increases the chance of injuries, more so in matches than in training sessions. Meanwhile, younger (amateur) players presented a higher incidence of injuries in training than in official matches. 23 Moreover, groups with an age range closer to the professional category suffered more injuries than the others. For instance, a sub-20 player group had injury rates similar to professional players, especially in matches than in training, while the sub-17 group had a higher injury rate during training. 24 This relationship is justified by the higher number of matches in the calendars of older age groups, which also happens in the transition from youth categories to professional soccer.

This information demonstrates that the load evolution during the athlete's progress to the professional category, if not balanced, has a strong relationship with the occurrence of injuries caused by the high physical demand in their training routine and matches.

#### Main Injuries

Injuries in soccer players, whether amateurs or professionals, mainly occur in the lower limbs. A study with male athletes competing in a regional soccer tournament investigated the incidence and prevalence of injuries and found that the lower limbs are more affected than other parts of the body, representing 86.9% of injuries. 6 Another author had consistent results, noting that 85.5% of the identified injuries affected the same anatomical region. 5 The lower limbs are the most required in soccer, explaining the higher frequency of injuries in this area. 8

The most common injury type includes muscular strains, mostly affecting the semitendinosus, biceps femoris, and gastrocnemius. 6 9 10 11 Strain results from fiber stretching beyond the physiological limits. It occurs during extravagant muscle contractions, such as rapid acceleration and deceleration, predisposing the player to an increased injury rate, with excess muscle tone as a major risk factor. 10

Although with a lower incidence, studies also mentioned other injuries, including sprains, contractures, and ligament ruptures. A sprain is a traumatic ligament injury affecting joint ligaments, particularly the ankle. In this injury, the main ligaments involved include the deltoid, anterior talofibular, and posterior talofibular. This type of trauma results from sudden movements, as small, repetitive injuries due to excessive activity can increase ligament fragility. The mechanism of this injury mainly results from plantar flexion during foot inversion, leading to a complete or partial rupture of the lateral ligaments. 12 13 Knee sprains are also common in soccer due to valgus torsion, that is, when the knee is directed sharply to the inside and the foot remains fixed. In this case, in addition to ligament lesions, meniscus injuries may occur. 14 15

Contracture results from an incorrect muscle contraction with no return to its regular relaxation state. Contractures occur due to lactic acid accumulation from anaerobic respiration after excessive overload. 16 Muscles damaged by this injury include the semitendinosus, semimembranosus, and biceps femoris. Soccer players are more susceptible to contractures, especially in the quadriceps, posterior thigh, gastrocnemius, and soleus, mainly from fatigue. 9 16 17 29

Ligament tears depend on the performed movement and mainly affect the anterior and posterior cruciate ligament. Anterior cruciate ligament injury, for instance, is common in athletes due to knee rotation with a fixed foot (facilitated by shoe cleats) and valgus stress in a deceleration movement, abrupt directional change, or both. 18 19 This injury can disrupt knee integrity and functionality in the long term and be irreversible; therefore, it accounts for many early retirements of soccer players. 20 The anterior talofibular ligament suffers injuries by excessive lateralization when walking on uneven surfaces.

#### FIFA11+ Prevention Program

The FIFA11+ prevention program had significantly better outcomes than the regular warm-up. As functional capacity influences the risk of injuries, athletes under 15 years old are more susceptible to them because of their lower physical development; as a result, a high physical demand can overload these players. 25

FIFA11+ considerably reduced injuries in commonly affected areas, including the knee, leg, and ankle. A special reduction occurred in contact, non-contact, and excessive use injuries. 26

As for professional players, FIFA11+ led to less impressive outcomes due to the competition calendar, short breaks between matches, and the highly physically demanding routine. However, it improved variables critical for good performance, such as the athlete's dynamic postural control. 27

## Final Considerations

The high physical demand influences an increase in the occurrence of muscle and ligament injuries in professional soccer players. Considering functional capacity, the increased intensity in the transition between base and professional categories, and the amount of training and competition calendars of official matches in professional soccer are relevant factors. The lower limbs are the most affected, and the most prevalent injuries include strains, sprains, contractures, and ligament ruptures.

Given the propensity for soccer injuries, it is critical to implement preventive interventions to care for the athletes' health. In this sense, we recommend FIFA 11+ or another customized method developed by the medical department of each club, taking into account individual features. These programs may reduce collective performance losses and health expenses.

Further studies are required to address the effects of the exacerbated routine of professional athletes and improve the performance and physical health of each player to increase the success in preventing injuries.
