# Supplementary material for: The Influence of High Physical Demand on the Occurrence of Major Muscle and Ligament Injuries in Professional Soccer Athletes: A Systematic Review
Source: Rev Bras Ortop (Sao Paulo). 2024 May 13;59(4):e510–8. doi: 10.1055/s-0044-1786171 (PMC11374400; doi:10.1055/s-0044-1786171)
Supplement: Related texts

# Research article: A influência da alta demanda física na ocorrência das principais lesões musculares e ligamentares em atletas profissionais de futebol: Uma revisão sistemática

**Keywords:** atletas, futebol/lesões, músculo esquelético, traumatismos do joelho

## Abstract

**Objetivo** Esta é uma revisão sistemática que objetivou investigar a influência da alta demanda física no aumento de lesões musculares e ligamentares em atletas profissionais de futebol.

**Métodos** Tratou-se de buscar, por meio da análise de publicações em meios científicos, as incidências das principais lesões, abordando suas causas e mecanismos bem como sua relação com a alta demanda física. Comparamos jogadores amadores (categorias de base) com jogadores profissionais e avaliamos a eficácia do FIFA11+ como alternativa de prevenção. As buscas foram realizadas nas plataformas Scielo, Pubmed e Google Scholar. Os filtros foram de acordo com o tema, data de publicação (últimos 5 anos) e relevância para o estudo. Foram utilizados os seguintes termos de indexação: *Overuse, Calendar, Injuries, Muscular, Ligament, Athletes, Soccer, Football* . Os principais dados obtidos foram descritos, objetivando a comparação e análise dos resultados. Foram aderidas as recomendações da declaração de Principais Itens para Relatar Revisões Sistemáticas e Metanálises *(Preferred Reporting Items for Systematic Reviews and Meta-Analyses* , PRISMA).

**Resultados** A pesquisa resultou em 24 artigos, publicados entre 2019 e 2023. Quanto à influência da alta demanda física, observou-se o aumento do risco de lesões. Em relação à incidência, constatou-se a prevalência de lesões nos membros inferiores. Quanto aos tipos de lesões, nota-se que as mais comuns são estiramento, entorse, contratura e rompimento ligamentar. Quanto ao FIFA11 + , apresentou-se como alternativa viável de prevenção.

**Conclusão** Concluiu-se que a alta demanda física aumenta a ocorrência das lesões musculares e ligamentares mais frequentes em futebolistas profissionais, que são estiramento, entorse, contratura e rompimento ligamentar, sugerindo-se o programa FIFA11+ como prevenção.

## Introdução

O futebol está entre os esportes com maior popularidade mundial, sendo praticado em diversos países, por indivíduos de todas as faixas etárias e ambos os sexos. Todavia, esta modalidade esportiva é acometida por um grande número de lesões em atletas profissionais, devido à intensa exigência física pela alta demanda nos treinamentos e calendário de jogos ao longo do ano. 1 2

A incidência de lesões desportivas e seus fatores de risco têm sido bastante discutida. A busca pelo bom desempenho e sucesso no esporte faz com que muitos indivíduos se submetam às condições de esforço físico limítrofe ou além de seus limites fisiológicos, resultando em lesões decorrentes da sobrecarga. 3 O futebol, neste contexto, é um esporte que exige muito impacto por contato físico, movimentos curtos, rápidos e descontínuos como aceleração e desaceleração, saltos e mudanças bruscas de direção. 1 4

Estima-se que o futebol é responsável por 50 a 60% das lesões esportivas e estas, podem vir a acometer diversos grupos musculares, ocorrendo em sua maioria, nos membros inferiores. 2 As injúrias, em geral, tem impacto negativo não somente na qualidade de vida do jogador, mas também na performance da equipe, resultando em prejuízos estratégicos e econômicos ao clube devido ao afastamento dos futebolistas em treinos e partidas. 5

O cuidado com a saúde do atleta e as estratégias de prevenção contra lesões são de extrema importância, pois além de maximizar as chances de sucesso da equipe, também otimiza o crescimento profissional do jogador. Portanto, o conhecimento acerca das lesões surge como estratégia para elaboração de planos que buscam prevenir ou minimizar no contexto esportivo, reduzindo os prejuízos e infelicidades que decorrem do afastamento dos jogadores. 6 7

Em virtude do crescimento de competições nacionais e internacionais, principalmente com o aumento da quantidade de jogos oficiais, torna-se indispensável o conhecimento acerca dos diversos tipos de lesões que mais acometem os jogadores de futebol, buscando principalmente a profilaxia destas, bem como o cuidado com a saúde e segurança do atleta. 6 Portanto, o presente estudo obteve como objetivo, investigar e abordar a influência da alta demanda física na ocorrência das principais lesões musculares e ligamentares em atletas profissionais de futebol.

## Materiais e Métodos

O presente trabalho foi conduzido conforme as diretrizes estabelecidas para a categoria de revisão sistemática, a qual possui a finalidade de examinar e discutir os artigos publicados sobre determinado assunto. 8 Foram aderidas as recomendações da declaração de Principais Itens para Relatar Revisões Sistemáticas e Metanálises *(Preferred Reporting Items for Systematic Reviews and Meta-Analyses* , PRISMA).

O presente estudo consiste de uma revisão sistemática. A revisão foi realizada em seis etapas: 1) identificação do tema e seleção da questão norteadora da pesquisa; 2) estabelecimento de critérios para inclusão e exclusão de estudos e busca na literatura; 3) definição das informações a serem extraídas dos estudos selecionados; 4) categorização dos estudos; 5) avaliação dos estudos incluídos na revisão integrativa e interpretação e 6) apresentação da revisão.

Na etapa inicial, para definição da questão de pesquisa foi utilizada a estratégia PICO (acrônimo para *Patient* , *Intervention* , *Comparation* e *Outcome* ). Assim, definiu-se a seguinte questão central que orientou o estudo: “A alta demanda física influencia no aumento da ocorrência das principais lesões musculares e ligamentares em atletas profissionais de futebol?” Nela, observa-se o P: atletas profissionais de futebol; I: alta demanda física; C: demanda física regular; O: aumento da ocorrência.

Para responder a esta pergunta, foi realizada a busca de artigos envolvendo o desfecho pretendido utilizando as terminologias cadastradas nos Descritores em Ciências da Saúde (DeCSs) criados pela Biblioteca Virtual em Saúde, desenvolvido a partir do *Medical Subject Headings da U.S. National Library of Medicine* , que permite o uso da terminologia comum em português, inglês e espanhol. Os descritores utilizados foram *: Overuse, Calendar, Injuries, Ligament, Muscular, Athletes, Football, Soccer* . Para o cruzamento das palavras chaves utilizou-se os operadores booleanos “ *and* ”, “ *or* ” e “ *not* ”.

Realizou-se um levantamento bibliográfico por meio de buscas eletrônicas nas seguintes bases de dados: *Scientific Electronic Library Online (SciELO), National Library of Medicine (PubMed)* e *Google Scholar* .

A busca foi realizada no mês de setembro de 2023. Como critérios de inclusão, não houve limitação de idiomas, sendo coletados artigos em inglês e português, publicados nos últimos 5 anos (2023–2019), que abordassem o tema pesquisado e que estivessem disponíveis eletronicamente em seu formato integral. Foram excluídos os artigos que não obedeceram aos critérios de inclusão.

## Resultados

Foram identificados 315 estudos no total e, ao aplicar o filtro de tempo de intervalo (2019–2023), restou um total de 157, os quais foram apurados, de acordo com os critérios de exclusão estabelecidos, resultando em 54 publicações a serem lidas integralmente. Destas, 30 foram excluídas por não apresentarem interesse ou não possuírem informações relevantes a serem tratadas no presente estudo. Por fim, foram selecionados 24 artigos para integrar esta revisão. O fluxograma ( Fig. 1 ) abaixo demonstra como foi realizado o processo de seleção citado.

**Fig. 1:** Fluxograma do processo de seleção dos artigos.

Os estudos selecionados foram tabelados ( Tabela 1 ) 5 6 8 9 10 11 12 13 14 15 16 17 18 19 20 21 22 23 24 25 26 27 28 29 , considerando o nome do autor e ano de publicação, tipo de artigo, título, objetivo do estudo e achados.

**Tabela 1:** Artigos utilizados para revisão sistemática

N°	Autor e ano	Tipo de artigo	Título	Principais achados
1	Drummond et al., 6 2021	Estudo prospectivo	Incidência de lesões em jogadores de futebol-mappingfoot: um estudo de coorte prospectivo	Em um torneio de futebol, 86,9% das lesões constatadas em competidores do sexo masculino foram nos membros inferiores. As lesões de estiramento são as mais comuns e acometem, principalmente, os músculos semitendíneo, bíceps femoral, gastrocnêmio, entre outros.
2	Marques, 5 2021	Revisão sistemática	Lesões em atletas portuguesas de futebol feminino	85,5% das lesões identificadas em atletas de futebol feminino foram nos membros inferiores.
3	Lima et al., 8 2022	Revisão sistemática	Lesões em atletas de futebol de campo: um estudo teórico	Lesões nos membros inferiores ocorrem com maior frequência no futebol, em razão da exigência desta região anatômica neste esporte.
4	Castelo et al., 9 2022	Revisão sistemática	Lesões no futebol profissional: uma revisão sistemática	As lesões de estiramento são as mais comuns e acometem, principalmente, os músculos semitendíneo, bíceps femoral, gastrocnêmio, entre outros. A contratura muscular prejudica os músculos semitendíneo, semimembranoso e bíceps femoral. A fadiga é a sua principal causa e, por essa razão, futebolistas de elite tendem a obter esse tipo de lesão.
5	Silva et al., 10 2019	Revisão sistemática	Incidência de lesões musculoesqueléticas em jogadores de futebol profissional no Brasil	As lesões de estiramento são as mais comuns e acometem os músculos semitendíneo, bíceps femoral, gastrocnêmio, entre outros. Compreende-se estiramento como o alongamento excessivo das fibras e ocorre por meio de movimentos de aceleração ou desaceleração brusca, ou, os *sprints.*
6	Teixeira et al., 11 2021	Pesquisa de campo	Análise epidemiológica de lesões em atletas profissionais de futebol em dois clubes de futebol de Goiânia-GO	As lesões de estiramento são as mais comuns e acometem os músculos semitendíneo, bíceps femoral, gastrocnêmio, entre outros.
7	Allah, 12 2022	Revisão sistemática	Fisioterapia nas lesões de entorse de tornozelo em jogadores de futebol: uma revisão de literatura	A entorse é uma lesão nos ligamentos de uma articulação, sendo comum no tornozelo, com possível ruptura parcial ou completa. Sua ocorrência se dá pelos movimentos bruscos, como a flexão plantar associada à inversão do pé.
8	Ribeiro et al., 13 2022	Revisão sistemática	A abordagem fisioterapêutica na entorse lateral de tornozelo: uma revisão de literatura.	A entorse é uma lesão nos ligamentos de uma articulação, sendo comum no tornozelo, com possível ruptura parcial ou completa. Sua ocorrência se dá devido a movimentos bruscos, como a flexão plantar associada à inversão do pé.
9	Meneses, 14 2021	Revisão sistemática	Lesões desportivas em atletas de futsal: uma revisão sistemática da literatura	No futebol, comumente observa-se entorses de joelho que ocorrem após mudança brusca de direção do joelho, mas com o pé fixo. Destaca-se as lesões capsuloligamentares e meniscais.
10	Feitoza Neto et al., 15 2021	Revisão sistemática	Lesões das estruturas do joelho em jogadores de futebol	No futebol, comumente observa-se entorses de joelho que ocorrem após mudança brusca de direção do joelho, mas com o pé fixo. Destaca-se as lesões capsuloligamentares e meniscais.
11	Farias, 16 2021	Revisão sistemática	Aplicabilidade dos exercícios de força na prevenção e reabilitação das lesões musculares grau I e II em jogadores de futebol de elite	A contratura decorre da contração incorreta do músculo, não retornando ao estado normal de relaxamento. Devido à sobrecarga de respiração anaeróbica, há acúmulo de ácido lático, causador deste fenômeno. A contratura muscular prejudica principalmente os músculos: semitendíneo, semimembranoso e bíceps femoral. A fadiga é sua principal causa e, por isto, futebolistas de elite costumam obter esse tipo de lesão.
12	Zhang and Wanh, 17 2023	Pesquisa de campo	Sports injuries in professional soccer players	A contratura muscular prejudica os músculos semitendíneo, semimembranoso e bíceps femoral. A fadiga é sua principal causa e, por isto, futebolistas de elite costumam obter esse tipo de lesão.
13	Silva et al., 18 2020	Revisão sistemática	Atuação do fisioterapeuta com jogadores que tiveram lesões no ligamento cruzado anterior	Os ligamentos podem romper de acordo com a forma que o movimento é executado. Em atletas, é comum observar lesões no ligamento cruzado anterior em função da força excessiva aplicada durante a rotação do joelho.
14	Borba, 19 2021	Revisão sistemática	Critérios de retorno ao futebol após a cirurgia de reconstrução do ligamento anterior: uma revisão de literatura	Os ligamentos podem romper de acordo com a forma que o movimento é executado. Em atletas, é comum observar lesões no ligamento cruzado anterior em função da força excessiva aplicada durante a rotação do joelho.
15	Sousa, 20 2021	Revisão sistemática	Pliometria na prevenção de lesão de ligamento cruzado anterior em atletas de futebol: uma revisão integrativa de literatura	Lesões de ligamento no joelho podem ocasionar perda de integridade e funcionalidade em curto e longo prazo, ou de maneira irreversível, sendo determinante para a aposentadoria de profissionais do futebol.
16	Choriyev, 21 2021	Pesquisa de Campo	Planning and Organizing Training of Football Players	É necessário considerar a capacidade funcional relativamente baixa de crianças de 8 a 11 anos envolvidas em esportes. Recomenda-se treinar essas crianças em técnicas de bola e táticas de jogo específicas para a idade. Para adolescentes de 12 a 17 anos, o foco muda para a preparação individual, com exercícios mais elaborados, com ênfase em velocidade de movimento, resistência, agilidade e flexibilidade em diferentes faixas etárias. Logo, é importante a seleção adequada de exercícios com base no conteúdo do jogo, influência sobre as funções do organismo, duração e intensidade dos exercícios.
17	Jones et al., 22 2019	Revisão Sistemática	Injury Incidence, Prevalence and Severity in High-Level Male Youth Football: A Systematic Review	A incidência total de lesões em atletas é influenciada pela idade. Atletas mais velhos tem maiores taxas de incidência de lesões. Um quinto das lesões ocorridas nos atletas são classificadas como graves e podem ocasionar afastamento de pelo menos 28 dias do atleta, o que prejudica o atleta e o time, uma vez que ambos perdem no desenvolvimento sazonal.
18	Pfirrmann et al., 23 2016	Revisão Sistemática	Analysis of Injury Incidences in Male Professional Adult and Elite Youth Soccer Players: A Systematic Review	As taxas de lesões foram maiores nos jogos do que nos treinos, tanto para jogadores jovens, como para adultos. Os jogadores juvenis tiveram maior incidência de lesões de treinamento do que os profissionais.
19	Cezarino et al., 24 2020	Estudo prospectivo	Injury Profile in a Brazilian First-Division Youth Soccer Team: A Prospective Study	A incidência de lesões foi maior nos jogos do que nos treinos, e o grupo etário mais velho (sub-20) obteve a maior taxa de incidência de lesões nos jogos, enquanto o grupo sub-17 obteve a maior taxa de incidência de lesões nos treinos. As taxas de incidência de lesões de jogadores menores de 20 anos aproximam-se de jogadores profissionais.
20	Yang et al., 25 2022	Revisão sistemática e meta-análise	Effects of the “FIFA11+ Kids” Program on Injury Prevention in Children: A Systematic Review and Meta-Analysis	O FIFA11+ diminuiu significativamente o risco de lesões gerais em comparação com o treino normal de aquecimento. Jogadores de futebol com menos de 15 anos correm o maior risco de lesões entre todas as faixas etárias analisadas pois possuem menor nível de desenvolvimento físico. O FIFA11+ Kids é eficaz na prevenção de lesões relacionadas com o futebol em jogadores jovens. O programa pode diminuir significativamente o risco de lesões no tornozelo, joelho e membros inferiores.
21	Al Attar et al., 26 2022	Ensaio clínico	The FIFA 11+ Kids Injury Prevention Program Reduces Injury Rates Among Male Children Soccer Players: A Clustered Randomized Controlled Trial	O FIFA11+ levou a uma redução significativa na ocorrência de lesões no joelho, perna e tornozelo, reduziu a probabilidade de um jogador se lesionar e também as taxas de lesões por contato, sem contato e por uso excessivo. Logo, esse estudo ratifica a importância da implementação do programa em jogadores mais novos com o intuito de prevenir as lesões.
22	Pinto et al., 27 2021	Revisão sistemática	FIFA 11+ para Prevenção de lesões em jogadores de futebol: revisão de revisões	Além de todas as indicações do estudo de Al Attar: O efeito de prevenção é superior quando todos os jogadores da equipe aderem ao programa. Quando direcionado a atletas de alta competição, os resultados são menos expressivos devido à periodização e ao calendário competitivo. Ainda, a chegada de jogadores de outros clubes no meio da época diminui a efetividade do programa, assim como os jogadores que deixam de realizar os protocolos por lesão ou doença. Ademais, o programa está também associado a melhoria nas variáveis de performance, como melhorias significativas no controle postural dinâmico.
23	Materne et al., 28 2020	Estudo prospectivo	Injury incidence and burden in a youth elite football academy: a four-season prospective study of 551 players aged from under 9 to under 19 years	Em comparação com outras faixas etárias, jogadores sub-16 e sub-18 obtiveram maiores taxas de incidência de lesões por sobrecarga. No restante de jogadores, contusões, entorses e lesões relacionadas ao crescimento foram os mais comuns. Lesões de menisco/cartilagem foram maior gravidade de lesão.
24	Aiello et al., 29 2022	Revisão sistemática	Injury-Inciting Activities in Male and Female Football Players: A Systematic Review	Atividades de corrida e chutes de alta intensidade podem ser as principais atividades desencadeantes de lesões na coxa e virilha, enquanto os duelos podem ser as atividades desencadeantes mais comuns de lesões no tornozelo. Atividades de duelo e pressão podem ser as atividades incitantes mais comuns que levam a lesões do ligamento cruzado anterior, mas não há um consenso na literatura.

Dos 24 artigos selecionados, 16 trabalhos foram revisões sistemáticas, 3 pesquisas de campo, 3 estudos prospectivos, 1 revisão sistemática e meta-análise e 1 ensaio clínico. No entanto, todos contemplam a temática principal: ocorrência das lesões esportivas em atletas de futebol. Dentre os principais objetivos dos autores estão: investigar a prevalência dos diferentes tipos de lesões, buscando as mais comuns; investigar as diferentes incidências de lesões em jogadores das categorias de base (considerados amadores neste estudo) e profissional; averiguar a eficácia do programa FIFA11+ como alternativa para prevenção de lesões; compreender a influência dos fatores de risco; compreender os mecanismos para ocorrência de determinados tipos de lesão.

## Discussão

## Sobre a Influência da Alta Demanda Física

## Na Capacidade Funcional

A capacidade funcional dos atletas é o primeiro ponto levado em consideração, uma vez que há diferença significativa entre atletas de categorias de base. Nesse quesito, considera-se necessárias diferentes estratégias de treinos para cada faixa etária, variando entre as categorias o nível de carga, a intensidade da atividade física, a duração dos exercícios assim como a seleção adequada destes.

A estratificação da modalidade esportiva e das variáveis de cada atleta proporciona indicações, como técnicas de bola e táticas de jogo, para atletas entre 8 e 11 anos, e preparação individual com ênfase em velocidade de movimento, resistência, agilidade e flexibilidade para aqueles atletas de 12 a 17 anos. Logo, evidencia-se a importância da gradação da intensidade, conforme o avançar da idade, com o intuito de preservar a saúde física do atleta até o nível profissional. Caso contrário, a falta de planejamento, com demandas exageradas, poderá expor esses jogadores à maiores chances de lesões. 21

## Comparação entre as Categorias de Base (Amadores) e Profissional

Foi analisada a taxa de incidência de lesões no futebol juvenil de alto nível e houve a descoberta que a ocorrência dessas lesões é influenciada pelo avançar da idade, visto que atletas mais velhos possuem maiores taxas de incidência, sendo 20% dessas lesões consideradas graves, ocasionado o afastamento do atleta de, pelo menos, 28 dias. 22

Essa análise condiz com o fato de o calendário de jogos da categoria profissional ser mais competitivo e exigir maior demanda física dos jogadores, quando comparado com o calendário dos atletas das categorias de base, considerados como amadores neste estudo. A maior frequência de pequenos intervalos entre jogos oficiais, a intensidade dos treinamentos, as variações de fuso-horário que afetam o descanso, a divergência de climas das variadas localidades e outros detalhes geram um conjunto de conjunto de fatores que contribuem para o maior desgaste físico do atleta profissional.

Os atletas mais novos que pertencem às categorias de base possuem um calendário menos intenso, gerando maiores chances de prevenção de lesões musculares e ligamentares por sobrecarga, sendo uma das justificativas da diferença nas incidências. Entretanto, observou-se que entre essa população de atletas, como nas categorias sub-9 até sub-19, o último grupo ainda obteve altas taxas de incidência de lesões, fato explicado pelo aumento da intensidade dos treinamentos e da frequência de jogos de campeonatos ao avançar da idade. Portanto, entende-se que a intensidade e a frequência de jogos crescentes, sem o devido preparo dos atletas, são fatores de risco para a ocorrência de lesões mesmo em atletas jovens. 28

Ademais, esse dado demonstra que a transição da categoria amador (categorias de base) para a profissional, seguindo a mesma noção do avançar da idade, poderá ser considerada um fator de risco para maiores ocorrências de lesões nesses atletas.

## Nos Treinamentos e Partidas

A alta demanda física em atletas de alta performance concentra maiores chances de lesões nos jogos do que nos treinos, enquanto os jogadores juvenis, considerados amadores neste estudo, obtiveram maior incidência de lesões no treinamento do que em jogos oficiais. 23 Outra observação é que os grupos com faixa etária mais próxima da categoria profissional sofreram mais lesões que os demais. Exemplo desse cenário ocorreu com um grupo sub-20 que obteve taxas de lesões muito próximas das taxas dos jogadores profissionais, sendo mais frequentes nas partidas do que nos treinamentos, enquanto o grupo sub-17 obteve maior taxa em treinamentos. 24 Essa relação justifica-se pela maior quantidade de jogos nos calendários de grupos etários superiores, o que também acontece na transição das categorias de base para o futebol profissional.

Essas informações demonstram que a evolução da carga durante o progresso do atleta para categoria profissional, se não balanceada, tem forte relação com a ocorrência de lesões provocadas pela alta demanda física em sua rotina de treinos e partidas.

## Sobre as Principais Lesões

Observou-se que as lesões em jogadores de futebol, amadores ou profissionais, concentram-se principalmente nos membros inferiores. Um estudo com atletas do sexo masculino que competem um torneio regional de futebol buscou investigar a incidência e prevalência de lesões e constatou que os membros inferiores são mais acometidos em relação às demais partes do corpo, representando um total de 86,9%. 6 Outro autor também obteve resultado semelhante em sua pesquisa, obtendo um total de 85,5% das lesões identificadas, acometidas na mesma região anatômica. 5 Os membros inferiores são mais requisitados neste esporte e, por este motivo, as lesões nessa área são mais frequentes. 8

Quanto aos tipos de lesões, observou-se que as mais comuns são as musculares de estiramento, acometendo músculos como: semitendíneo, bíceps femoral, gastrocnêmico, entre outros. 6 9 10 11 O estiramento é caracterizado pelo alongamento das fibras, superior aos limites fisiológicos. Este fato ocorre durante contrações musculares extravagantes, como em episódios de rápida aceleração e desaceleração, predispondo o jogador de futebol ao risco aumentado para este tipo de complicação, sendo o excesso de tônus muscular um grande fator de risco. 10

Embora com menor incidência, outras lesões também foram mencionadas nos estudos, dentre estas: entorse, contraturas e rompimento ligamentar. A entorse é uma lesão ligamentar traumática que acomete os ligamentos de uma articulação, com maior prevalência no tornozelo, sendo os principais ligamentos envolvidos: deltoide, talofibular anterior e talofibular posterior. Este tipo de trauma ocorre devido a movimentos bruscos, em que pequenas lesões de repetição por excesso de atividade podem predispor a uma maior fragilidade desses ligamentos. O mecanismo deste tipo de lesão se dá, principalmente, devido ao movimento de flexão plantar em associação à inversão do pé, o que consequentemente leva a uma ruptura, seja parcial ou completa, dos ligamentos laterais. 12 13 Entorses de joelho também são comumente observadas no futebol, principalmente quando em torção em valgo, isto é, quando o joelho é direcionado bruscamente para o lado interno e o pé permanece fixo. Neste caso, além dos ligamentos, lesões de menisco são potenciais. 14 15

A contratura ocorre quando o músculo contrai de forma incorreta e não regressa ao estado normal de relaxamento. Isto acontece em função do acúmulo de ácido lático que resulta da respiração anaeróbica após sobrecarga excessiva. 16 Dentre os músculos prejudicados por este tipo de lesão estão: o semitendíneo, o semimembranoso, o bíceps femoral e outros. Os jogadores de futebol estão mais suscetíveis a este tipo de lesão, principalmente no quadríceps, posterior da coxa, gastrocnêmio e sóleo, e um dos principais motivos que levam a sua ocorrência é a fadiga. 9 16 17 29

Quanto aos rompimentos ligamentares, estes podem sofrer ruptura a depender da execução de um movimento (ligamento cruzado anterior e ligamento cruzado posterior, principalmente). A lesão do ligamento cruzado anterior, por exemplo, é comumente observada em atletas, devido à rotação do joelho com a fixação do pé (facilitada pelas travas das chuteiras) e estresse em valgo em movimento de desaceleração e/ou mudança de direção abrupta. 18 19 Este tipo de lesão pode gerar desordem na integridade e funcionalidade do joelho à longo prazo ou ainda, sem reversão, sendo, portanto, responsável por muitas aposentadorias precoces de jogadores de futebol. 20 Já o ligamento talofibular anterior é lesionado pela lateralização excessiva nas pisadas em superfícies irregulares.

## Sobre o Programa de Prevenção FIFA11+

Observou-se que o programa de prevenção FIFA11+ obteve resultados significativamente superiores ao aquecimento comum. Como a capacidade funcional é um dos fatores levados em consideração no risco de lesões, atletas com menos de 15 anos de idade são mais suscetíveis à ocorrência de lesões, uma vez que o desenvolvimento físico é menor e uma alta demanda física pode sobrecarregar esses atletas. 25

Os locais comumente acometidos, tais como joelho, perna e tornozelo, sofreram uma considerável redução de lesões com o programa de prevenção, sendo as lesões por contato, sem contato e por uso excessivo reduzidas. 26

Quanto aos atletas profissionais, o FIFA11+ obteve um resultado menos expressivo, em virtude do calendário competitivo, com intervalos curtos entre as partidas, além da rotina com alta exigência física. Entretanto, notou-se a melhora em variáveis que são consideradas fundamentais para a boa performance, como o controle postural dinâmico do atleta. 27

## Considerações Finais

A alta demanda física influencia no aumento da ocorrência de lesões musculares e ligamentares em atletas profissionais de futebol, considerando a capacidade funcional, o aumento da intensidade na transição entre categorias de base e categoria profissional e a quantidade de treinos e calendários competitivos de jogos oficiais no futebol profissional como fatores relevantes. Os membros inferiores são os mais acometidos, destacando-se, com maior prevalência, o estiramento, a entorse, a contratura e o rompimento ligamentar.

Diante da propensão à ocorrência de lesões neste esporte, é de suma importância a implementação de intervenções preventivas que visem o cuidado com a saúde corporal do atleta. Neste sentido, sugere-se a utilização do FIFA 11+ ou outro método personalizado, desenvolvido pelo departamento médico de cada equipe, atendendo suas individualidades. Assim, é possível diminuir prejuízos no desempenho coletivo da equipe e gastos com saúde.

Por fim, faz-se necessário mais estudos e investigações que abordem os efeitos da rotina exacerbada dos atletas profissionais, visando melhorar o desempenho e saúde física de cada um destes, a fim de obter maiores sucessos nas prevenções de injúrias.
